# Virus Genotype-Dependent Transcriptional Alterations in Lipid Metabolism and Inflammation Pathways in the Hepatitis C Virus-infected Liver

**DOI:** 10.1038/s41598-019-46664-0

**Published:** 2019-07-22

**Authors:** W. M. H. d’Avigdor, M. A. Budzinska, M. Lee, R. Lam, J. Kench, M. Stapelberg, S. V. McLennan, G. Farrell, J. George, G. W. McCaughan, T. Tu, N. A. Shackel

**Affiliations:** 1 Liver Injury and Cancer Laboratory, Centenary Institute of Cancer Medicine and Cell Biology, Sydney, NSW Australia; 2 Sydney Medical School, University of Sydney, Sydney, NSW Australia; 3 Gastroenterology and Liver Research Laboratory, Ingham Institute, Liverpool, NSW Australia; 4 Australian National University, Canberra, ACT Australia; 5 Storr Liver Centre, Westmead Institute for Medical Research, University of Sydney and Westmead Hospital, Westmead, NSW 2145 Australia; 6 A.W. Morrow Gastroenterology and Liver Centre, Royal Prince Alfred Hospital, Sydney, NSW Australia; 7 Department of Infectious Diseases, Molecular Virology, Heidelberg University Hospital, Heidelberg, Germany; 8 South-Western Sydney Clinical School, University of New South Wales, Liverpool, NSW Australia; 9 Department of Gastroenterology and Hepatology, Liverpool Hospital, Liverpool, NSW Australia; 10 German Center for Infection Research (DZIF), Heidelberg Partner Site, Heidelberg, Germany

**Keywords:** Hepatitis C virus, Hepatitis C, Liver cirrhosis, Microarrays

## Abstract

Despite advances in antiviral therapy, molecular drivers of Hepatitis C Virus (HCV)-related liver disease remain poorly characterised. Chronic infection with HCV genotypes (1 and 3) differ in presentation of liver steatosis and virological response to therapies, both to interferon and direct acting antivirals. To understand what drives these clinically important differences, liver expression profiles of patients with HCV Genotype 1 or 3 infection (n = 26 and 33), alcoholic liver disease (n = 8), and no liver disease (n = 10) were analysed using transcriptome-wide microarrays. In progressive liver disease, HCV genotype was the major contributor to altered liver gene expression with 2151 genes differentially expressed >1.5-fold between HCV Genotype 1 and 3. In contrast, only 6 genes were altered between the HCV genotypes in advanced liver disease. Induction of lipogenic, lipolytic, and interferon stimulated gene pathways were enriched in Genotype 1 injury whilst a broad range of immune-associated pathways were associated with Genotype 3 injury. The results are consistent with greater lipid turnover in HCV Genotype 1 patients. Moreover, the lower activity in inflammatory pathways associated with HCV genotype 1 is consistent with relative resistance to interferon-based therapy. This data provides a molecular framework to explain the clinical manifestations of HCV-associated liver disease.

## Introduction

Hepatitis C virus (HCV) is a common blood-borne virus that infects about 180 million people worldwide^1^. Chronic HCV infection occurs in 80% of exposed individuals and causes progressive liver disease that can leads to cirrhosis and hepatocellular carcinoma (HCC)^2^. HCV isolates are separated into seven genotypes, including (in order of prevalence) Genotype 1 (~46% of chronic HCV infections), 3 (~22%), 2 (~13%) and 4 (~13%)^1,3^.

Two common genotypes, HCV genotype 1 (G1) and 3 (G3), are associated with different clinical presentations, including severity of hepatic steatosis and in their responses to both interferon (IFN) and direct acting antiviral (DAA) therapy. In individuals with HCV G3 infection, hepatic steatosis has been reported to be more severe and directly associated with viral load^4,5^, with significant improvement in steatosis upon sustained viral clearance^6,7^. Further, serum cholesterol levels are decreased in individuals infected with HCV G3 compared to other HCV genotypes^8^, consistent with virus-induced dysregulation of lipid metabolism. On the other hand, metabolic (as opposed to virus-induced) steatosis tends to predominate in individuals infected with HCV G1 infection.

Individuals infected with different HCV genotypes also vary in their response to interferon-based therapies. Traditional combined therapy using interferon and ribavirin is successful in only ~50% of individuals infected with HCV G1, compared with ~80% in HCV G3^9–11^. Moreover, with new highly-effective direct acting antiviral treatments (NS3/4 A protease inhibitors in particular), patients infected with HCV G3 are more likely to result in virological failures^12–14^. Thus, understanding the underlying molecular pathways that lead to the phenotypic differences between HCV genotypes may improve treatment success.

The molecular mechanisms underlying these inter-genotypic differences is not clear. Therefore, we set out to compare the liver transcriptomes of patients chronically infected by different HCV genotypes. We hypothesise that differences in observed clinical features (in particular the differences in hepatic steatosis and interferon response) could be attributed down to specific altered regulatory networks or functional pathways in the host response to HCV. Thus, we used whole transcriptome microarrays to characterise the expression profiles of liver tissue obtained from individuals infected with either HCV G1 or G3 and used rigorous bioinformatics analysis to identify key altered cellular pathways.

## Results

### Extensive HCV genotype-dependent expression alterations are observed in progressive liver disease prior to the development of advanced cirrhosis

**Figure 1:**
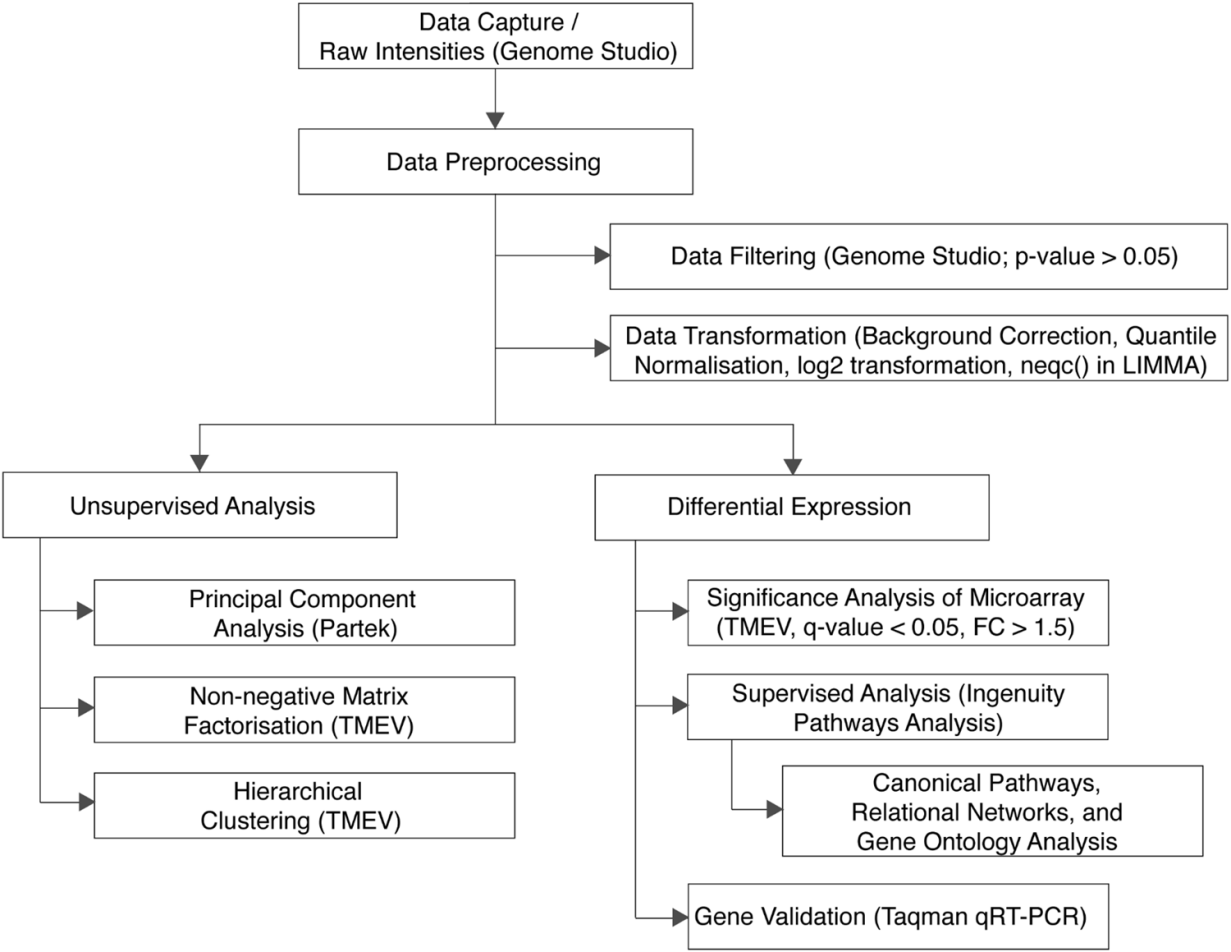
Microarray analysis workflow. Workflow outlining the microarray analysis workflow including data processing and downstream analysis.

In total, liver tissues from 26 patients infected with HCV G1 and 33 with HCV G3, 3 with an unknown HCV genotype, 8 with alcoholic liver disease, and 10 non-diseased liver control specimens were analysed by microarray analysis. Patient characteristics (including fibrosis grade, inflammation grade and steatosis) are shown in Table 1. Patients were divided into two groups: the progressive liver injury (n = 45), in which patients had normal or well-compensated liver function; and the advanced liver injury (n = 36), in which patients had advanced cirrhosis many with decompensated disease. In total, 26,427 probes (14,875 genes) were expressed in at least one sample (p < 0.05, t-test) after background subtraction and were used for downstream analyses (Fig. 1).

**Table 1:** Patient demographics.

Characteristic		NDL (n = 6)	HCV G1 (n = 16)	HCV G3 (n = 23)	G3 vs G1 p-value
Progressive Liver Injury					
Age (years)		17.5 ± 3.7 (13–22)	46.6 ± 8.1 (30–59)	41.0 ± 4.6 (33–49)	p = 0.008
Male/Female		3/1*	8/8	23/0	
Fibrosis stage			1.31 ± 1.4	2.48 ± 1.3	p = 0.011
0		6 (100%)	6 (38%)	2 (9%)	
1			5 (31%)	3 (13%)	
2			1 (6%)	8 (35%)	
3			2 (12%)	2 (9%)	
4			2 (12%)	8 (35%)	
Portal/Periportal inflammation grade			1.94 ± 0.8)	2.4 ± 0.7	ns
0		6 (100%)	0 (0%)	0 (0%)	
1			5 (31%)	2 (9%)	
2			7 (44%)	11 (48%)	
3			4 (25%)	9 (39%)	
4			0 (0%)	1 (4%)	
Steatosis			0.81 ± 0.8	1.2 ± 0.7	ns
0		6 (100%)	7 (44%)	3 (13%)	
1			5 (31%)	13 (57%)	
2			4 (24%)	6 (26%)	
3			0 (0%)	1 (4%)	
Advanced Liver Injury					
Characteristic	NDL (n = 4)	HCV G1 (n = 10)	HCV G3 (n = 11)	non-G1/G3 (n = 3)	ALD (n = 8)
Cirrhosis (HCC)		5 (50%)	6 (55%)	3 (100%)	3 (37%)
Cirrhosis (Explant)		5 (50%)	5 (45%)		5 (63%)

*gender was unavailable for 3 anonymous non-diseased liver samples;

Mean ± SD (range); NDL: non-diseased liver; ns: not significant.

**Figure 2:**
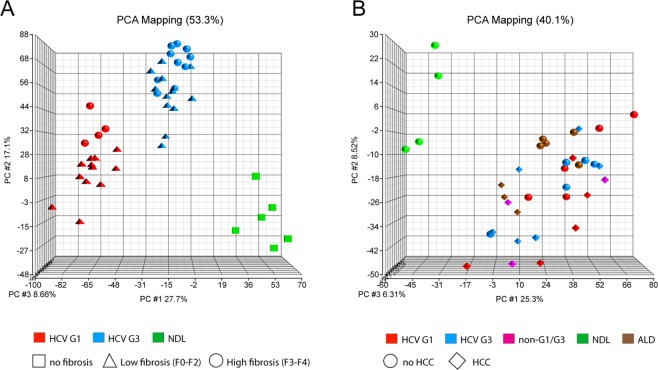
Principal Component Analysis of the whole liver gene expression profiles of HCV-induced progressive liver injury (**A**) and advanced liver injury (**B**). Based on the first three principal components, three major clusters are apparent in progressive liver injury: patients infected with HCV genotype 1 (red), genotype 3 (blue) and non-diseased liver (green). Secondary clustering based on low fibrosis (F0–F2; triangles) and high fibrosis (F3–F4, circles) was also observed. In advanced HCV liver injury, only clustering based on general liver injury (but not on HCV genotype) was observed.

In the progressive liver injury group, viral genotype determined the greatest difference in gene expression. Principal component analysis and non-negative matrix factorisation identified three groups: HCV G1-infected, HCVG3-infected, and uninfected control samples (Fig. 2A and Supplementary Fig. 1A). Within these three main groups, samples also show secondary clustering based on fibrosis grade. Hierarchical clustering showed a similar result based on global transcript expression (Supplementary Fig. 1C). Interestingly, in the advanced liver injury group, HCV genotype-dependent clustering was not observed, but instead all samples (regardless of HCV genotype or injury aetiology) cluster together as a large group, away from the non-diseased control samples (Fig. 2B, Supplementary Fig. 1B,D). This suggests that the liver expression profile of advanced cirrhotic patients converges, despite the different initial hepatic transcriptional expression in earlier stages of chronic HCV infection.

**Figure 3:**
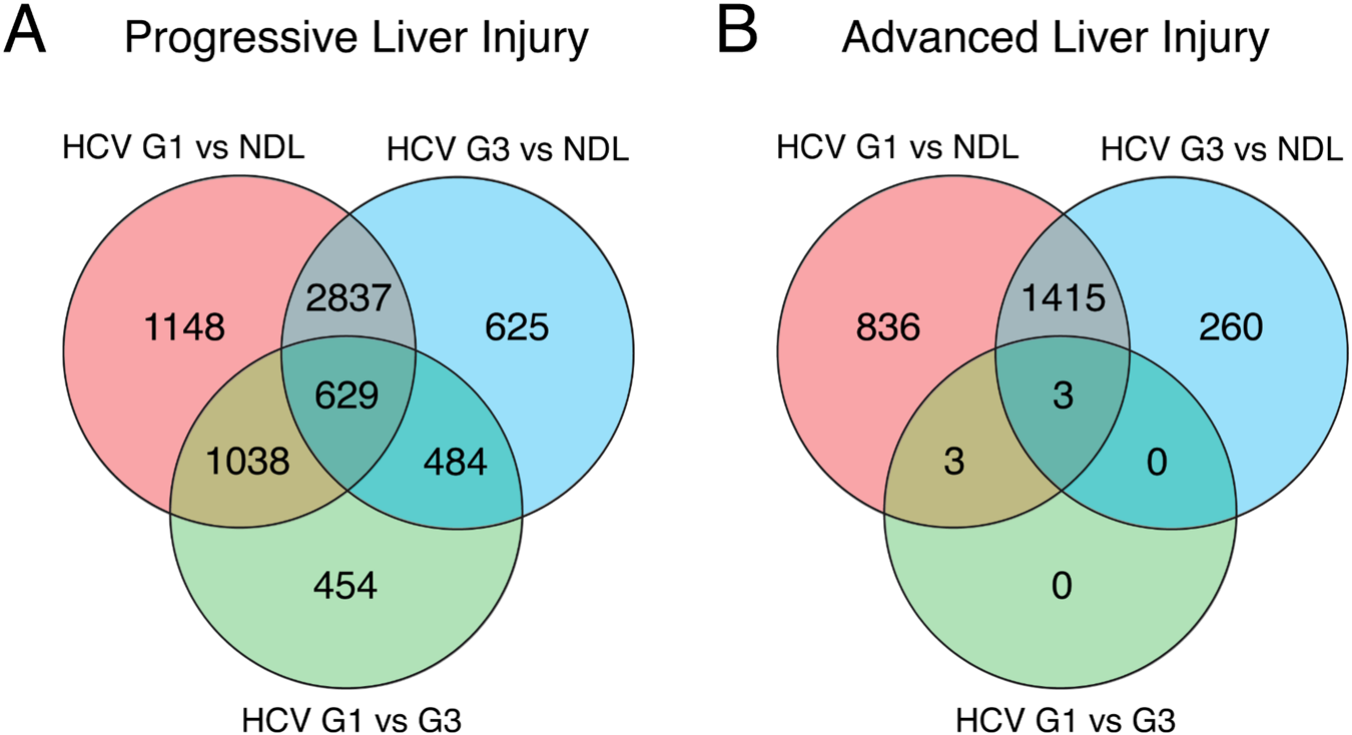
Comparative differential gene expression between non-diseased livers (NDL) and livers from patients infected with HCV genotype 1 (HCV G1) or genotype 3 (HCV G3). Venn diagrams show the overlap of differentially expressed liver genes in HCV-induced progressive (**A**) and advanced (**B**) liver injury.

The difference in HCV-genotype dependent expression alterations between progressive and advanced liver disease was further observed in the number of differentially expressed genes. As expected, we observed a large number of genes differentially expressed between disease and non-diseased samples (Fig. 3). A total of 6761 genes (45.5% of all genes expressed) had a >1.5-fold altered expression in HCV-infected samples compared to non-diseased liver, with the majority (4610, or 31% of all genes expressed) not significantly different between HCV genotypes. These included (among others) interferon stimulator genes, as expected. Indeed, we confirmed upregulation of two example ISGs RSAD2 and ISG15 by Taqman quantitative PCR (Supplemental Fig. 2), consistent with the microarray analysis.

A comparison of differentially expressed liver transcripts in HCV G1 and HCV G3 infection showed a total of 2605 genes (17,5% of all genes expressed, top 100 genes listed in Supplemental Table 1) were significantly and differentially expressed by >1.5-fold in the progressive liver disease group (Fig. 3A). In marked contrast, in the advanced cirrhotic patients there were only 6 differentially expressed genes observed at >1.5-fold difference between HCV G1 and HCV G3 infections (Fig. 3B). Importantly these 6 transcripts were all interferon stimulated genes that were upregulated in HCV G1-infected samples. These differences between progressive and advanced liver disease cohorts remained when we increased the differential expression threshold to >2-fold difference or reduced it (Supplemental Fig. 3). In summary, the relatively large number of differentially expressed genes between HCV G1 and G3 infections in progressive liver injury suggests that HCV genotypes induce liver disease through different interactions with host pathways. However, inter-genotypic differences are no longer observed in advanced liver injury, during which liver gene expression changes are dominated by the extent of injury.

### Lipid metabolism-associated regulatory gene networks are altered with HCV genotype during progressive liver injury

We undertook a functional analysis, inferred from the transcript differences shown between genotypes, described in progressive but not advanced HCV liver injury. We used Ingenuity Pathway Analysis (IPA, Ingenuity Systems, Mount View, CA) to analyse the genes differentially expressed >1.5-fold between the two HCV genotypes. We found gene regulatory networks associated with lipid metabolism were the most highly significant altered between the HCV G1 and G3 (Table 2).

**Table 2:** Genotype-specific regulatory gene networks altered in progressive HCV liver injury.

Diseases and functions gene network	Ratio*	−log_10_ (p-value)^^^
HCV genotype 3 vs genotype 1		
Lipid Metabolism, Molecular Transport, Small Molecule Biochemistry (A)	18/33	8
Cellular Development, Cellular Growth and Proliferation, Organ Development (A)	4/7	2
HCV genotype 1 vs NDL		
Lipid Metabolism, Small Molecule Biochemistry, Vitamin and Mineral Metabolism (A)	35/35	13
Lipid Metabolism, Molecular Transport, Small Molecule Biochemistry (B)	17/22	2
Cellular Development, Cellular Growth and Proliferation, Organ Development (B)	6/8	1
Cell Death and Survival, Gastrointestinal Disease, Hepatic System Disease	4/6	1
HCV genotype 3 vs NDL		
Lipid Metabolism, Small Molecule Biochemistry, Vitamin and Mineral Metabolism (B)	15/17	5
Lipid Metabolism, Small Molecule Biochemistry, Vitamin and Mineral Metabolism (C)	16/25	2
Lipid Metabolism, Molecular Transport, Small Molecule Biochemistry (C)	14/25	1
Cellular Development, Cellular Growth and Proliferation, Organ Development (C)	5/7	1
Cancer, Cell Death and Survival, Organismal Injury and Abnormalities	3/4	1

*Ratio: The number of differentially expressed genes (up- or down-regulated), when compared with the total number of genes within a specified canonical pathway, as defined by Ingenuity Pathways Analysis; ^^^Score = −log_10_(p-value); only those networks with at least three focus molecules, as well as a −log_10_(p-value) ≥1 in the network are included in the analysis. Letters refer to a unique network with the same gene ontology.

**Figure 4:**
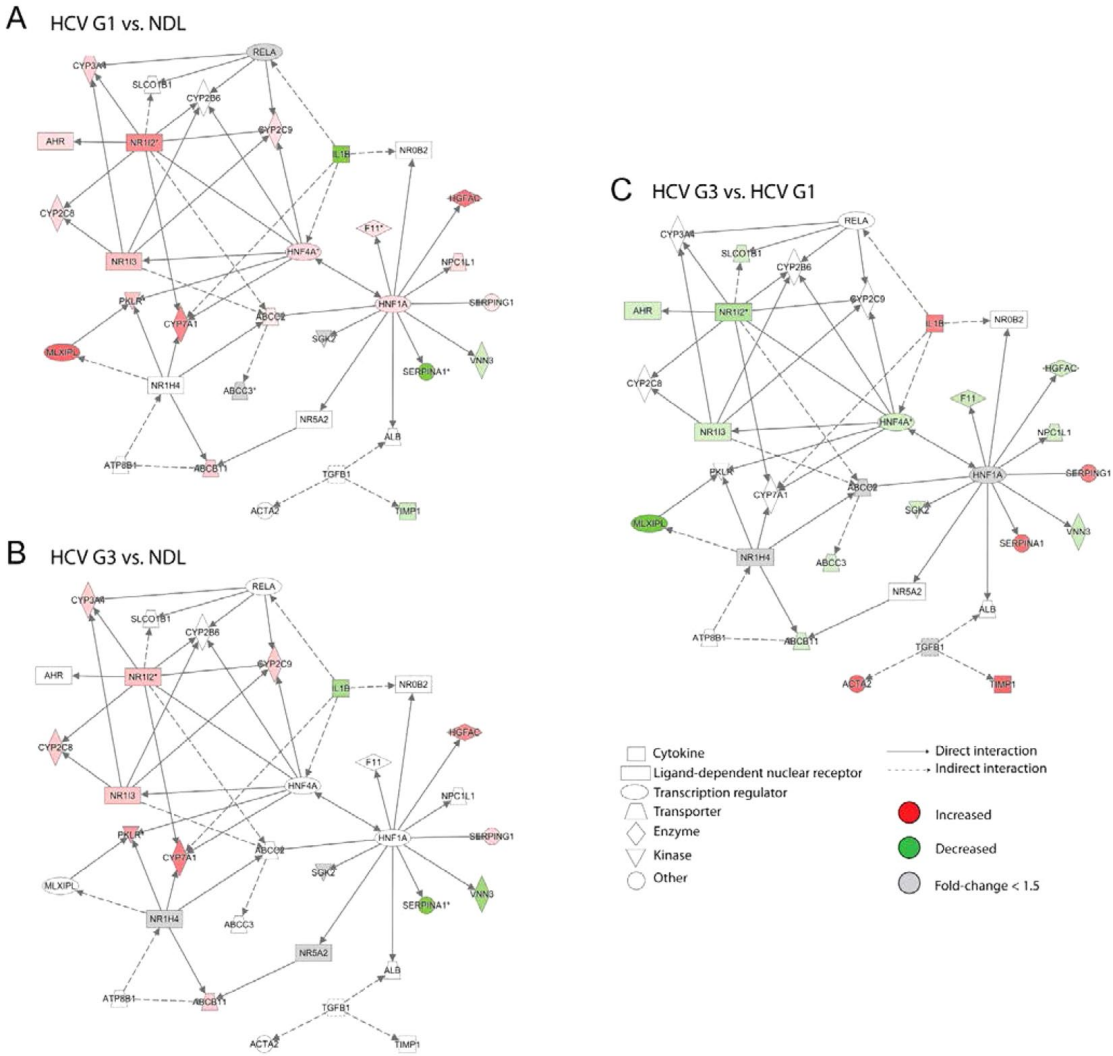
Differentially expressed genes in the ‘Lipid Metabolism, Molecular Transport, Small Molecule Biochemistry’ pathway. Expression data overlayed onto these networks was visualised for differentially expressed genes between HCV genotype 1 (HCV G1) compared to non-diseased liver (NDL) (**A**); HCV genotype 3 (HCV G3) compared to NDL (**B**); and HCV G1 compared to HCV G3 (**C**). Node colours corresponds to fold changes of differentially expressed genes (red = up-regulated; green = down-regulated; grey = genes not meeting the significance threshold of −1.5< fold change <1.5).

**Figure 5:**
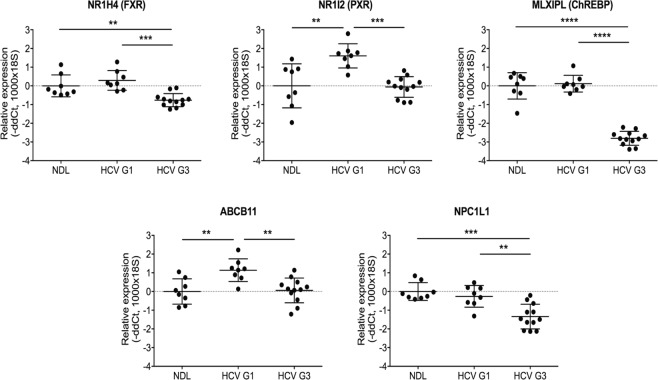
Validation of the observed differentially expressed genes in HCV genotype 1- and 3-infected liver tissues. Taqman qRT-PCR validation of selected genes in lipid metabolism pathway analysis of progressive HCV-induced liver disease, comparing non-diseased liver (NDL, n = 8) and livers infected with HCV genotype 1 (HCV G1, n = 8) or genotype 3 (HCV G3, n = 12). Mann-Whitney *U*-test; **p < 0.01, ***p < 0.001, ****p < 0.0001.

We visualised this enriched lipid metabolism network to determine associations with HCV injury and genotype (Fig. 4). Particularly interesting regulatory genes that had increased expression in HCV G1-infected livers (compared to non-diseased liver controls) included nuclear receptors NR1I2 (also known as PXR), and the transcriptional regulators HNF4A, HNF1A, and MLXIPL (also known as ChREBP) (Fig. 4A). These regulatory genes were not increased to the same extent in HCV G3-infected livers (Fig. 4B,C) consistent with genotype-specific alterations in these networks. HCV genotype-specific transcriptional alterations of several of these regulatory genes (FXR, PXR, and ChREBP) and their downstream genes (ABCB11 which is upregulated by FXR; NPCL1 which is upregulated by HNF1A) were confirmed by Taqman quantitative PCR, validating the observations from the microarray analysis (Fig. 5).

### HCV genotype-dependent alterations in fatty acid degradation, cholesterol transport and immune mediators

The transcript expression was subject to IPA analysis to further identify specific functional pathways significantly altered comparing HCV G1 and G3-infected livers and found high representation of two general categories: lipid/cholesterol-metabolism and immune/inflammation-associated pathways (Table 3).

**Table 3:** Genotype-specific canonical pathways altered in progressive HCV liver injury.

Ingenuity Canonical Pathways	Genes (n)	HCV G3 vs HCV G1			HCV G1 vs NDL			HCV G3 vs NDL		
		Rank	−log(p-value)	Ratio(%)*	Rank	−log(p-value)	Ratio(%)*	Rank	−log(p-value)	Ratio(%)*
Leukocyte Extravasation Signaling	117	**1**	**4.73**	**32.5**	—	—	—	333	0.30	32.5
Agranulocyte Adhesion and Diapedesis	84	**2**	**3.45**	**32.1**	—	—	—	75	1.18	40.5
Granulocyte Adhesion and Diapedesis	91	**3**	**3.21**	**30.8**	—	—	—	33	1.69	42.9
MSP-RON Signaling Pathway	27	**4**	**3.18**	**44.4**	—	—	—	—	—	—
B Cell Receptor Signaling	113	**5**	**2.89**	**28.3**	—	—	—	—	—	—
Role of Tissue Factor in Cancer	70	**6**	**2.77**	**31.4**	255	0.33	40	45	1.41	42.9
Role of Pattern Recognition Receptors in Recognition of Bacteria and Viruses	76	**7**	**2.63**	**30.3**	270	0.30	39.5	240	0.52	35.5
Glioma Invasiveness Signaling	39	**8**	**2.53**	**35.9**	245	0.35	41	210	0.61	38.5
Natural Killer Cell Signaling	56	**9**	**2.48**	**32.1**	—	—	—	192	0.63	37.5
FcƟ Receptor-mediated Phagocytosis in Macrophages and Monocytes	57	**10**	**2.39**	**31.6**	—	—	—	36	1.64	45.6
Bile Acid Biosynthesis, Neutral Pathway	13	—	—	—	**1**	**3.01**	**84.6**	**2**	**2.92**	**76.9**
Triacylglycerol Biosynthesis	21	264	0.32	19	**2**	**2.60**	**71.4**	350	0.27	33.3
Noradrenaline and Adrenaline Degradation	21	—	—	—	**3**	**2.60**	**71.4**	58	1.36	52.4
Serotonin Degradation	37	—	—	—	**4**	**2.06**	**59.5**	**9**	**2.33**	**54.1**
Coagulation System	31	60	1.21	29	**5**	**2.03**	**61.3**	14	2.13	54.8
LPS/IL-1 Mediated Inhibition of RXR Function	158	265	0.32	17.1	**6**	**1.99**	**48.1**	**5**	**2.61**	**43**
LXR/RXR Activation	99	228	0.41	18.2	**7**	**1.94**	**50.5**	78	1.12	39.4
PXR/RXR Activation	56	—	—	—	**8**	**1.76**	**53.6**	15	2.06	48.2
NAD biosynthesis II (from tryptophan)	9	133	0.75	33.3	**9**	**1.68**	**77.8**	125	0.90	55.6
Stearate Biosynthesis I (Animals)	25	—	—	—	**10**	**1.59**	**60**	143	8.35	44
Virus Entry via Endocytic Pathways	54	90	0.98	24.1	294	0.26	38.9	**1**	**3.51**	**55.6**
Endothelin-1 Signaling	103	217	0.45	18.4	12	1.56	48.5	**3**	**2.83**	**46.6**
Macropinocytosis Signaling	42	115	0.82	23.8	—	—	—	**4**	**2.70**	**54.8**
Prolactin Signaling	53	292	0.27	17	103	0.69	45.3	**6**	**2.47**	**50.9**
Thrombopoietin Signaling	39	196	0.49	20.5	109	0.66	46.2	**7**	**2.39**	**53.8**
Xenobiotic Metabolism Signaling	183	236	0.38	17.5	121	0.61	41.5	**8**	**2.35**	**41.5**
Hepatic Cholestasis	116	86	0.99	21.6	100	0.71	43.1	**10**	**2.31**	**44**

*Ratio: The percentage of differentially expressed genes (up- or down-regulated), when compared with the total number of genes within a specified canonical pathway, as defined by Ingenuity Pathways Analysis, -log10(p-value) <1.30; -, not applicable.

Analysis of regulatory pathways showed both lipogenic and lipolytic pathways were activated in HCV G1-infected livers. In the pathways altered between HCV-infected and non-diseased livers, enrichment was seen in genes associated with TAG biosynthesis in HCV G1-infected livers. Triacylglycerol biosynthesis pathways (though higher than non-diseased livers) were expressed at significantly lower levels in tissues infected with HCV G3. This is consistent with fatty acid synthesis is induced at a higher level in HCV G1 (vs. G3) infection.

Interestingly, no significant differences in gene expression were found to be associated with varying levels of steatosis (Brunt steatosis score of 2 or 3 when compared with 0 or 1, data not shown). Further, when analysing the disease and bio-functions associated with the genes differentially expressed between the HCV genotypes (Table 4), gene ontologies associated with cerebrotendinous xanthomatosis (a genetic disease associated with dysregulated metabolism and storage of lipids, cholesterol, and bile acids), primary biliary cirrhosis, transport of bile salt, biliary excretion of cholesterol, and excretion of lipid were observed. This is consistent with an increased export of cholesterol and bile salts in individuals infected with HCV G1.

**Table 4:** Genotype-specific diseases and biofunctions altered in HCV-induced progressive liver injury.

Diseases or Functions Annotation	−log_10_(p-value)	Genes (n)
**HCV genoptype 3 vs HCV genotype 1**		
colorectal cancer	3.11	30
abdominal cancer	2.78	471
digestive system cancer	2.78	471
metastatic colorectal cancer	2.41	27
neoplasia of epithelial tissue	2.40	455
hepatocellular carcinoma	2.36	454
liver tumor	2.34	455
primary biliary cirrhosis	2.34	6
transport of bile salt	2.06	4
Budd-Chiari syndrome	1.90	6
biliary excretion of cholesterol	1.55	2
proliferation of oval cells	1.55	2
uptake of organic anion	1.55	2
excretion of lipid	1.45	3
**HCV genotype 1 vs NDL**		
cerebrotendinous xanthomatosis	2.47	6
excretion of lipid	2.06	5
progressive intrahepatic cholestasis	2.00	28
damage of liver	1.86	10
progressive familial intrahepatic cholestasis type 1	1.83	27
injury of liver	1.59	9
liver adenoma	1.45	5
**HCV genotype 3 vs NDL**		
metastasis	3.00	47
metastatic colorectal cancer	2.94	46
colorectal cancer	2.77	47
progressive intrahepatic cholestasis	1.86	24
cerebrotendinous xanthomatosis	1.82	5
progressive familial intrahepatic cholestasis type 1	1.67	23
Budd-Chiari syndrome	1.55	8
biliary excretion of lipid	1.48	3
excretion of lipid	1.40	4
damage of liver	1.33	8

−log_10_(p-value) < 1.30.

In inflammation pathways, the gene signature enriched in HCV G3 (compared to G1) infection is associated with both the innate immune and the adaptive immune response (Table 3). The leading 10 canonical pathways that were upregulated in HCV G3 compared to G1 were all immune associated pathways. Moreover, the analysis of HCV-genotype dependent genes in their associated disease and bio-functions (Table 4) shows enrichment of various injury or cancer gene ontologies as well as those associated with activation of differentiation liver stem cell compartments (e.g., proliferation of oval cells). The activation of these pathways (likely associated with chronic inflammatory microenvironment) appeared to be more enriched in HCV G3 compared to HCV G1 infected livers. Interestingly, the level of interferon signalling response (as measured by ISG expression) appeared to be lower in HCV G3 (compared to G1) infection (Supplementary Fig. 2), suggesting a HCV genotype-dependent attenuation of these pathways.

Clinical studies have shown an association between HCV G3 and accelerated progression of fibrosis^5,15^. Therefore, we specifically analysed HCV genotype-associated transcript expression in the context of fibrosis associated pathways and observed both pro-fibrotic and anti-fibrotic genes were differentially-expressed in HCV G3 compared to G1 (Supplemental Table 2), with no obvious enrichment in either pathway. Fibrosis is a balance of pro- and anti-fibrosis factors^16,17^. Therefore, we are unable to determine if there is a net difference in fibrosis pathways between the HCV genotypes based on this data.

## Discussion

The described transcript changes in progressive injury, and the comparison with advanced injury, highlight a number of key and previously unrecognised aspects of the molecular pathogenesis of HCV liver injury. We found that the major determinant for altered transcription prior to advanced liver disease was viral genotype (comparing HCV G1 vs G3). Consistent with previous studies^15^, viral genotype played a greater role in determining host liver gene expression than fibrosis, inflammation and steatosis (prior to decompensated cirrhosis). Upon severe cirrhosis, we found that these HCV genotype-dependent alterations give way to a general liver injury response, with only ISGs being significantly different between the HCV genotypes (with HCV G3 showing significantly lower transcriptional levels compared to HCV G1 infected livers). Interestingly, the majority of the genotype-dependent alterations in early stages of liver disease progression were associated with lipid metabolism, suggesting an underlying mechanism for the increased liver steatosis severity observed in patients chronically infected with HCV G3.

Our results are agree with previous studies showing increased expression of ISGs, immune cell activity, and regulators of lipid metabolism upon HCV infection in chimpanzee infection models^16,17^, human biopsies^15,18,19^, and *in vitro* studies^20^. A key point revealed by the current study was the association of these transcriptional alterations with HCV genotype in progressive but not advanced cirrhotic liver disease. A number of studies have shown increased ISG transcript expression in HCV G1-infected livers^19,21,22^, but this is the first study to show that this genotype difference is only evident prior to end-stage liver disease. This suggests differing mechanisms for the development of HCV associated liver injury in HCV G1 vs G3 progressive injury that ultimately differs to the shared transcript expression seen in end stage injury. Transcript expression in early and progressive stages of liver injury are consistent with a generalised response to injury as well as dominant HCV genotype specific responses. As disease progresses and damage increases, the injury-associated (but not the viral-associated) response becomes dominant. Future work is needed to define these generalised liver damage responses over time: for example, it is unclear if patients with early stage injury would have progressed to advanced injury (unknown as these patients all received anti-HCV therapy following liver biopsy). For the current study, we take a conservative interpretation by remaining agnostic on whether the differences between HCV G1 and G3 are result of the stage of injury or if the expression of these pathways protects against the progression to advanced cirrhosis.

Upon focused analysis, we found no consistent genotype-dependent transcriptional differences in fibrosis associated-pathways. While some studies have linked HCV G3 infection with an accelerated fibrosis progression rate^5,15^, the majority of clinical studies (reviewed in^23^) have shown no association between HCV genotype and increased progression of fibrosis. Our study did not identify any overall changes associated with genotype-dependent fibrosis stage.

Consistent with previous studies, the transcriptional activation of immune/inflammatory pathways were shown to be HCV genotype-dependent. In particular, this data shows that inflammatory pathways are consistently higher in HCV G3 when compared with HCV G1 infections, as others have previously reported^19,24,25^. To what extent these transcriptional alterations are involved in the observed increased sensitivity of HCV G3 infections to interferon therapy^9^ is not known. Paradoxically, the present study also found that HCV G1 induces a higher (pre-treatment) ISG transcription, which has previously been associated with poorer interferon treatment responses^19^. Therefore HCV G1 appears to have an increased resistance to innate immune responses compared to HCV G3.

The steatosis grade of patients included in this study with HCV G1 and G3 infection was not significantly different overall. However, a greater percentage of patients had some steatosis in HCV G3 (87%) compared to HCV G1 (56%) infection. In HCV-associated steatosis there are both shared and distinct pathogenic pathways when comparing HCV genotypes. The pathways that are distinct between infection with different HCV genotypes may result in changes in lipid composition more than the grade of steatosis or the overall lipid amount. Multiple transcriptomic studies have shown increased transcription of lipid metabolism pathways in HCV infection^23,26–28^. This is not surprising as HCV replicates on lipid droplets^29^, and so is expected to upregulate pathways that increase their formation.

Importantly, given our larger cohorts compared to previous studies, we are first to show that these alterations are differentially altered depending on HCV genotype. In particular, we found that lipid metabolism-associated pathways (e.g., triacylglycerol biosynthesis, LXR/RXR Activation, and Stearate Biosynthesis) appear to be more highly induced in HCV G1 infection compared to G3 infection. Regulatory transcripts that were more highly expressed in HCV G1-infected livers are involved in increased lipolysis and prevention of insulin resistance and this may, in part explain, why HCV G3 patients have a more severe presentation of hepatic steatosis. An increase in ChREBP in HCV G1 infection is associated with maintaining insulin signalling sensitivity during fatty liver disease, induces expression of lipogenic enzyme acetyl-CoA carboxylase and fatty acid synthase genes. Conversely, hepatocyte-specific ChREBP knock-down and knock-out improves hepatic steatosis and insulin signalling in obese mice^30^ and induces synthesis of cholesterol (as opposed to fatty acids) from intracellular sugars (e.g. fructose)^31^. Additionally, PXR reduces lipolysis by downregulating fatty acid beta oxidation^32^. Further, the increased transcript expression of some of the gene regulatory networks in HCV G1-infected livers are associated with decreased steatosis. For example, knock-down of HNF4a induces fatty liver disease in normal mice by inhibiting secretion of VLDLs^33^. Similarly, hepatocyte deletion if HNF1a induces increased fatty acid synthesis and upregulation of inflammatory cytokines^34^.

It is possible that these genotype-dependent alterations in lipid-associated pathways are driven by interferon-dependent pathways (many ISGs are involved in lipid metabolism^35^), though it is unknown to what extent this occurs relative to ISG-independent mechanisms. Interestingly, pathways contributing to both intrahepatic lipid increase (e.g. fatty acid synthesis and cholesterol transport into the liver) and decrease (e.g. lipolysis and bile export) were enriched in HCV G1 livers^35^.

There is overlap between the annotated biological pathways described in our analyses. For example, the disease profiles in the biofunctions analysis recurrently calls up cancer-related pathways (Table 4). However, these pathways include many genes that can also be associated with liver turnover, inflammation, dedifferentiation and altered metabolism. There is a suggestion that HCV G3 is linked to higher rates of HCC (reviewed in^36^). However, the tissue used in this study is from the non-tumour liver that did not contain histologically-abnormal pre-neoplastic hepatocytes. Therefore, the interpretation of the results needs to account for the overlap especially in understanding HCV G3 related injury prior to the development of HCC.

**Figure 6:**
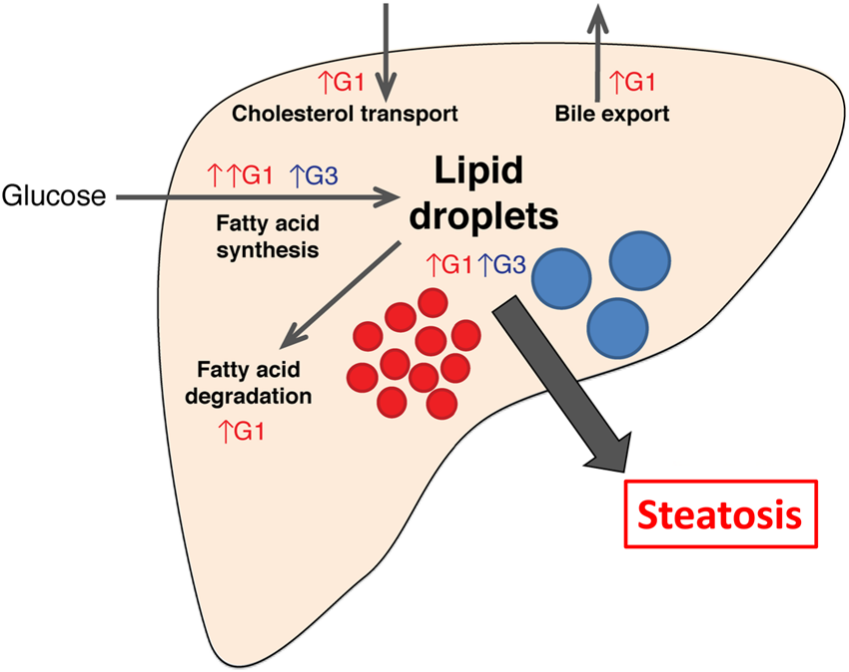
Summary of HCV genotype-specific lipid pathways. Gene expression analysis suggests that HCV G1 induces increased fatty acid degradation, bile acid transport, drug metabolism, and decreased cholesterol export, while these pathways are less altered in the HCV G3-infected liver. Activation of large numbers of lipid metabolism (both synthesis and degradation) genes may drive the continual turnover of lipid droplets in HCV G1 infection. In contrast, HCV G3 may simply induce an increase in size of existing lipid droplets. Though different in clinical presentation, both result in extensive intrahepatic steatosis.

Recent data of HCV liver biopsies showing no significant difference in intrahepatic lipid levels within liver biopsies of patients infected with either HCV G1 or G3^37^. However, in these studies, greater numbers of small lipid droplets were seen in tissues from HCV G1 patients, with the authors suggesting that this is a sign of increased lipolysis and lipid droplet turnover. Our results are consistent HCV G3 inducing altered lipid metabolism in such a way that the cell cannot adapt to the increased intracellular lipid load (e.g. blocking signalling feedback loops) (Fig. 6). Furthermore, hepatocytes infected with HCV G1 adapt to achieve homeostasis, arriving a new equilibrium where lipid formation and breakdown are balanced. This is consistent with a previous hypothesis that HCV G3 (but not HCV G2) selectively inhibits cholesterol synthesis pathways through a mechanism that overcomes the host’s ability to maintain homeostasis, thereby leading to the observed HCV-dependent hypocholesterolaemia^38^. Interestingly, if this were proven true in follow-up studies, it could provide a mechanism of why when the successful treatment of HCV G3 improves steatosis (by removing this virus-mediated block), while in HCV G1 infections maintain this new stable state regardless of HCV treatment outcome.

Our observational study is a cross-sectional liver-specific snapshot of HCV associated liver injury. However, lipid metabolism in the liver involves a complex interplay of intracellular molecular pathways, secreted cytokines and hormones, and multiple interacting organ systems (e.g. muscle, adipose, and pancreatic tissues). Environmental factors likely complicate matters further (e.g. patient diet leading to obesity and metabolic syndrome). Moreover, genetic factors also play a role, exemplified by some patients developing progressive injury while others tolerate chronic inflammation with limited disease progression. Finally, virus-host responses add complexity: virus factors alter host responses, which in turn the virus adapts to. To begin to understand the effectors of the HCV genotype-dependent changes that we have shown, an analysis of the acute phase of HCV infection (e.g. in cell culture and animal models) will likely be required. While liver transcriptome-wide studies have been carried out during the acute phase of HCV infection^16,39,40^, to our knowledge, different HCV genotypes have not been directly compared. The major limitation of human studies is the rarity of acute infection liver samples with chimpanzees being the only animal model susceptible to HCV infection. Further current *in vivo* approaches fail to account for human infection complexity (hepatoma cell line or primary hepatocyte mono-culture represents the vast majority of *in vitro* systems used in the field) and lack diversity (in general, the HCV genotype 2a JFH1 strain is used for the majority of *in vitro* infections).

In summary, this study has shown clear and major differences in the gene expression signatures (particularly of lipid metabolism and inflammation) between HCV G1 and G3 infections in the progressive stages of liver disease. The molecular mechanisms described include changes in pathways linked to lipid turnover and inflammation. Future studies are required to functionally characterise specific pathways. This will identify molecular pathways that could be targeted in HCV-associated steatosis and improve patient outcomes.

## Methods

### Patient sample acquisition

The Human Research Ethics Committee of the Royal Prince Alfred Hospital approved this study (Protocols X05-0343 and X10-0072). All methods were performed in accordance with the relevant guidelines and regulations. Informed and written consent was obtained from all patients. Non-tumour human liver tissues were collected at the Royal Prince Alfred Hospital (RPAH), Sydney, Australia. Explant liver tissues extraneous to clinical need at liver transplant and liver core biopsies were immediately snap frozen after collection and stored at −80 °C until processing.

Liver tissue was also taken for histopathology analysis in each case, fixed in 4% formaldehyde and assessed for grades of inflammation, fibrosis (using the Scheuer scoring system^41^) and steatosis (using the Brunt scoring system^42^) by Dr. James Kench from the Anatomical Pathology Department, RPAH. All clinical and histopathology information are summarised in Table 1.

### RNA extraction and quantitation

Snap frozen liver tissue (explants and biopsies) were processed by a tissue bead homogeniser (Qiagen, Hamburg, Germany) and RNA was extracted using either an RNAqueous® or RNAqueous®-Micro kit, followed by the DNA-*free*® kit (Thermofisher, Waltham, MA, USA). RNA extracts were assessed for purity using the NanoDrop 1000 (NanoDrop Technologies, Wilmington, CA, USA), and analysed for RNA integrity and quantified using the Bioanalyser 2100 (Agilent Technologies, Santa Clara, CA, USA). RNA extracts with an RNA integrity number (RIN) >6 were considered satisfactory for further analysis.

### Microarray analysis of mRNA transcripts

Total RNA (250 ng) was amplified to make cRNA using the Illumina® TotalPrep RNA amplification kit (Thermofisher, Waltham, MA, USA) and quantified using the Bioanalyser 2100. cRNA was hybridized to Illumina® whole genome Human Sentrix® BeadChip microarrays (Human-WG6 version 2) for 16 hours. Each BeadChip was scanned and read with the Illumina® BeadScan software in the Illumina® BeadArray Reader (Thermofisher, Waltham, MA, USA) and used to provide the summary of a raw intensity value for each probe on each array. Microarray data has been made available on ArrayExpress (Accession E-MTAB-7751).

Sample quality control and probe filtering was performed in GenomeStudio^®^ (version 1.1.1) using the probe annotation file HumanWG-6_V2_0_R4_11223189_A.bgx (48,701 probes or 18,414 known RefSeq genes). A signal-to-noise ratio of >10, equivalent to the ratio of the 95^th^ (P95) and 5^th^ (P05) percentiles of all pixel intensities, was considered satisfactory. Probes that did not have a significantly greater signal compared to background fluorescence in at least one sample across the entire dataset were excluded (t-test, p-value < 0.05). Raw summary expression data was then corrected for background intensity levels by normal exponential convolution using negative controls, log2 transformed, and quantile normalized using the neqc function in the LIMMA package from Bioconductor Software project^43,44^ in the R programming environment (http://www.r-project.org).

### Analysis overview

mRNA transcript microarray data were analysed by three main analyses (Fig. 1): unsupervised analysis to show global differences based on changes in gene expression; differential expression to determine specific genes that were expressed between known patient groups; and supervised analysis of the differentially expressed genes to determine the specific cellular pathways and functions affected.

### Unsupervised analysis

Principal component analysis of gene expression data was performed using Partek Genomic Studio^45^ (Partek, St Louis, OH, USA). Non-negative matrix factorization was used to reduce the dimension of gene expression data and identify distinct molecular patterns using the Brunet *et al*. algorithm^46^, with a Kullback Leibler divergence and random starting point to initialise the algorithm (seed = 123456). To get a robust estimate of the factorization rank (r = 2,3,4, and 5), 30 runs were performed with a maximum of 500 iterations. Hierarchical clustering was carried out using the Pearson correlation with average linkage method in Multiple Experiment Viewer (MeV) version 4.8.1 (The Institute for Genomic Research^47,48^).

### Differential expression

The significant analysis of microarrays (SAM) method was used to identify the genes that were differentially expressed between the groups using the MeV^46^, version 4.8.1. Each group was compared to the other groups using 1000 permutations, to protect against non-normal data distribution. The cut-off for significance, determined by the tuning parameter delta, was chosen based on the median false discovery rate (q-value) <1%, with a fold change threshold >1.5.

### Supervised analysis

The Ingenuity Pathway Analysis^49^ (IPA, Qiagen Inc., Redwood City, CA, USA) was used to identify the pathways, gene networks and disease/functions of the differentially expressed genes. The Core analysis was performed using setting with ‘Tissue and Cell Lines’ as ‘Liver’ and expression fold change cut-off 1.5. Fisher’s Exact test was used to calculate the p-value with the significance threshold p < 0.05.

### Quantitative reverse transcription polymerase chain reaction (qRT-PCR)

Reverse transcription was performed using Superscript III as per the manufacturer’s instructions (Thermofisher, Waltham, MA, USA). Each reaction comprised of 250 ng RNA in a volume of 20 µL, containing 500 nM dNTPs, 12.5 ng/µL random hexamers (Roche, Basel, Switzerland), 1x first strand buffer, 5 mM DTT, and 10 U/µL Superscript III, and RNase-free water to volume (Baxter, Deerfield, IL, USA). Reverse transcriptase reactions were carried out in a thermocycler at 25 °C for 5 minutes, 50 °C for 60 minutes, and 70 °C for 15 minutes. The resultant cDNA was stored at −20 °C until further use.

qRT-PCR was performed on the following genes using Taqman gene expression assays (Thermofisher, Waltham, MA, USA): Nuclear Receptor Subfamily 1 Group H Member 4 (NR1H4, Hs01026590_m1; also known as Farnesoid X-Activated Receptor, FXR), Nuclear Receptor Subfamily 1 Group I Member 2 (NR1I2, Hs01114267_m1; also known as Pregnane X Receptor, PXR), MLX Interacting Protein Like (MLXIPL, Hs00975714_m1; also known as Carbohydrate Response Element Binding Protein, ChREBP), ATP Binding Cassette Subfamily B Member 11 (ABCG11, Hs00184824_m1), ATP Binding Cassette Subfamily G Member 5 (ABCG5, Hs00223686_m1), NPC1 (Niemann-Pick Disease, Type C1, Gene)-Like 1 (NPC1L1, Hs00203602_m1), Radical S-Adenosyl Methionine Domain Containing 2 (RSAD2, Hs00369813_m1; also known as viperin), and Interferon-stimulated Gene 15 (ISG15); and normalised to the 18 S endogenous control (Hs99999901_s1, 6 replicates). Each qRT-PCR was reaction was in a total volume of 12.5 µL containing 1x Taqman primer and probe set, 1x universal Taqman mastermix, 2 µL cDNA (1:5 dilution), made up to volume with RNase-free water (Baxter, Deerfield, IL, USA); and was carried out in a Stratagene Mx3000P machine (Thermofisher, Waltham, MA, USA) at 50°C for 2 minutes, 95 °C for 10 minutes, and 45 cycles of 95 °C for 15 seconds, and 60 °C for 1 minute. qRT-PCR data was analysed using the ΔΔCt method^50^.

### Statistical analysis

Graph Pad Prism® (version 4.0.3) was used for statistical analysis. Two groups were compared using the Mann-Whitney *U*-test; and greater than two groups were compared using the Kruskal-Wallis test, with the Dunn’s correction.

## Supplementary information

Supplemental data

Supplementary Table S2

## References

[1] Gower E, Estes C, Blach S, Razavi-Shearer K, Razavi H (2014). Global epidemiology and genotype distribution of the hepatitis C virus infection. J Hepatol.

[2] Tu T, Buhler S, Bartenschlager R (2017). Chronic viral hepatitis and its association with liver cancer. Biol Chem.

[3] Sievert W (2011). A systematic review of hepatitis C virus epidemiology in Asia, Australia and Egypt. Liver Int.

[4] Rubbia-Brandt L (2000). Hepatocyte steatosis is a cytopathic effect of hepatitis C virus genotype 3. J Hepatol.

[5] Adinolfi LE (2001). Steatosis accelerates the progression of liver damage of chronic hepatitis C patients and correlates with specific HCV genotype and visceral obesity. Hepatology.

[6] Kumar D, Farrell GC, Fung C, George J (2002). Hepatitis C virus genotype 3 is cytopathic to hepatocytes: Reversal of hepatic steatosis after sustained therapeutic response. Hepatology.

[7] Castera L (2004). Effect of antiviral treatment on evolution of liver steatosis in patients with chronic hepatitis C: indirect evidence of a role of hepatitis C virus genotype 3 in steatosis. Gut.

[8] Corey KE (2009). Hepatitis C virus infection and its clearance alter circulating lipids: implications for long-term follow-up. Hepatology.

[9] Manns MP (2001). Peginterferon alfa-2b plus ribavirin compared with interferon alfa-2b plus ribavirin for initial treatment of chronic hepatitis C: a randomised trial. Lancet.

[10] Fried MW (2002). Peginterferon alfa-2a plus ribavirin for chronic hepatitis C virus infection. N Engl J Med.

[11] Hadziyannis, S. J. *et al*. Peginterferon-alpha2a and ribavirin combination therapy in chronic hepatitis C: a randomized study of treatment duration and ribavirin dose. *Ann Intern Med* **140**, 346–355, doi:140/5/346 (2004). 10.7326/0003-4819-140-5-200403020-00010 14996676

[12] Kwo PY (2017). Glecaprevir and pibrentasvir yield high response rates in patients with HCV genotype 1-6 without cirrhosis. J Hepatol.

[13] Tapper EB, Afdhal NH (2013). Is 3 the new 1: perspectives on virology, natural history and treatment for hepatitis C genotype 3. J Viral Hepat.

[14] Foster GR (2011). Telaprevir alone or with peginterferon and ribavirin reduces HCV RNA in patients with chronic genotype 2 but not genotype 3 infections. Gastroenterology.

[15] Chen L (2010). Cell-type specific gene expression signature in liver underlies response to interferon therapy in chronic hepatitis C infection. Gastroenterology.

[16] Su AI (2002). Genomic analysis of the host response to hepatitis C virus infection. Proc Natl Acad Sci USA.

[17] Bigger, C. B. *et al*. Intrahepatic gene expression during chronic hepatitis C virus infection in chimpanzees. *J Virol* **78**, 13779–13792, doi:78/24/13779 (2004). 10.1128/JVI.78.24.13779-13792.2004 PMC533929 15564486

[18] Asselah, T. *et al*. Liver gene expression signature to predict response to pegylated interferon plus ribavirin combination therapy in patients with chronic hepatitis C. *Gut* **57**, 516–524, doi:gut.2007.128611 (2008). 10.1136/gut.2007.128611 17895355

[19] Sarasin-Filipowicz M (2008). Interferon signaling and treatment outcome in chronic hepatitis C. Proc Natl Acad Sci USA.

[20] Helbig KJ, Lau DT, Semendric L, Harley HA, Beard MR (2005). Analysis of ISG expression in chronic hepatitis C identifies viperin as a potential antiviral effector. Hepatology.

[21] Robinson MW (2015). Viral genotype correlates with distinct liver gene transcription signatures in chronic hepatitis C virus infection. Liver Int.

[22] Holmes JA (2015). The relationships between IFNL4 genotype, intrahepatic interferon-stimulated gene expression and interferon treatment response differs in HCV-1 compared with HCV-3. Aliment Pharmacol Ther.

[23] Fujino, T. *et al*. Expression profile of lipid metabolism-associated genes in hepatitis C virus-infected human liver. *Hepatol Res* **40**, 923–929, doi:HEP700 (2010). 10.1111/j.1872-034X.2010.00700.x 20887597

[24] Dower K, Ellis DK, Saraf K, Jelinsky SA, Lin LL (2008). Innate immune responses to TREM-1 activation: overlap, divergence, and positive and negative cross-talk with bacterial lipopolysaccharide. J Immunol.

[25] Arts RJ, Joosten LA, van der Meer JW, Netea MG (2013). TREM-1: intracellular signaling pathways and interaction with pattern recognition receptors. J Leukoc Biol.

[26] Younossi, Z. M. *et al*. Gene expression profile associated with superimposed non-alcoholic fatty liver disease and hepatic fibrosis in patients with chronic hepatitis C. *Liver Int*, doi:LIV2060 (2009). 10.1111/j.1478-3231.2009.02060.x 19515216

[27] Ryan, M. C., Desmond, P. V., Slavin, J. L. & Congiu, M. Expression of genes involved in lipogenesis is not increased in patients with HCV genotype 3 in human liver. *J Viral Hepat*, doi:JVH1283 (2010). 10.1111/j.1365-2893.2010.01283.x 20196803

[28] Yasui K (2010). Steatosis and hepatic expression of genes regulating lipid metabolism in Japanese patients infected with hepatitis C virus. J Gastroenterol.

[29] Miyanari Y (2007). The lipid droplet is an important organelle for hepatitis C virus production. Nat Cell Biol.

[30] Dentin R (2006). Liver-specific inhibition of ChREBP improves hepatic steatosis and insulin resistance in ob/ob mice. Diabetes.

[31] Zhang D (2017). Lipogenic transcription factor ChREBP mediates fructose-induced metabolic adaptations to prevent hepatotoxicity. J Clin Invest.

[32] Huang YY, Gusdon AM, Qu S (2013). Nonalcoholic fatty liver disease: molecular pathways and therapeutic strategies. Lipids Health Dis.

[33] Yin L, Ma H, Ge X, Edwards PA, Zhang Y (2011). Hepatic hepatocyte nuclear factor 4alpha is essential for maintaining triglyceride and cholesterol homeostasis. Arterioscler Thromb Vasc Biol.

[34] Ni Q (2017). Deletion of HNF1alpha in hepatocytes results in fatty liver-related hepatocellular carcinoma in mice. FEBS Lett.

[35] de Veer MJ (2001). Functional classification of interferon-stimulated genes identified using microarrays. J Leukoc Biol.

[36] Goossens N, Negro F (2014). Is genotype 3 of the hepatitis C virus the new villain?. Hepatology.

[37] Campana B (2018). *In vivo* analysis at the cellular level reveals similar steatosis induction in both hepatitis C virus genotype 1 and 3 infections. J Viral Hepat.

[38] Clark PJ (2012). Hepatitis C virus selectively perturbs the distal cholesterol synthesis pathway in a genotype-specific manner. Hepatology.

[39] Boldanova T, Suslov A, Heim MH, Necsulea A (2017). Transcriptional response to hepatitis C virus infection and interferon-alpha treatment in the human liver. EMBO Mol Med.

[40] Bigger CB, Brasky KM, Lanford RE (2001). DNA microarray analysis of chimpanzee liver during acute resolving hepatitis C virus infection. J Virol.

[41] Scheuer PJ (1991). Classification of chronic viral hepatitis: a need for reassessment. J Hepatol.

[42] Brunt, E. M., Janney, C. G., Di Bisceglie, A. M., Neuschwander-Tetri, B. A. & Bacon, B. R. Nonalcoholic steatohepatitis: a proposal for grading and staging the histological lesions. *Am J Gastroenterol* **94**, 2467–2474, doi:S0002927099004335 (1999). 10.1111/j.1572-0241.1999.01377.x 10484010

[43] Ritchie ME, Dunning MJ, Smith ML, Shi W, Lynch AG (2011). BeadArray expression analysis using bioconductor. PLoS Comput Biol.

[44] Ritchie ME (2015). limma powers differential expression analyses for RNA-sequencing and microarray studies. Nucleic Acids Res.

[45] Yeung KY, Ruzzo WL (2001). Principal component analysis for clustering gene expression data. Bioinformatics (Oxford, England).

[46] Brunet JP, Tamayo P, Golub TR, Mesirov JP (2004). Metagenes and molecular pattern discovery using matrix factorization. Proc Natl Acad Sci USA.

[47] Loganantharaj, R., Cheepala, S. & Clifford, J. Metric for Measuring the Effectiveness of Clustering of DNA Microarray Expression. *BMC Bioinformatics* **7** Suppl 2, S5, doi:1471-2105-7-S2-S5 (2006). 10.1186/1471-2105-7-S2-S5 PMC1683560 17118148

[48] Saeed AI (2003). TM4: a free, open-source system for microarray data management and analysis. Biotechniques.

[49] Kramer A, Green J, Pollard J, Tugendreich S (2014). Causal analysis approaches in Ingenuity Pathway Analysis. Bioinformatics.

[50] Livak KJ, Schmittgen TD (2001). Analysis of relative gene expression data using real-time quantitative PCR and the 2(-Delta Delta C(T)) Method. Methods (San Diego, Calif.

