# Supplementary material for: Virus Genotype-Dependent Transcriptional Alterations in Lipid Metabolism and Inflammation Pathways in the Hepatitis C Virus-infected Liver
Source: Sci Rep. 2019 Jul 22;9:10596. doi: 10.1038/s41598-019-46664-0 (PMC6646375; doi:10.1038/s41598-019-46664-0)
Supplement: Supplementary file 1 — Supplemental data [file 41598_2019_46664_MOESM1_ESM.pdf]

Supplemental data for "Virus Genotype-Dependent Transcriptional Alterations in Lipid Metabolism and Inflammation Pathways in the Hepatitis C Virus-infected Liver"

d'Avigdor W.M.H.*[1,2], Budzinska M.A.*[1,2,3], Lee M. [1], Lam R. [1], Kench J.[2], Stapelberg M.[1,2], McLennan S.V.[2], Farrell G.[4], George J.[5], McCaughan G.W.[1,2,6], Tu T.[#1,7,8,10], and Shackel N.A. [#1,2,8,9]

* Authors contributed equally to this study; # Authors contributed equally to this study

[1] Liver Injury and Cancer Laboratory, Centenary Institute of Cancer Medicine and Cell Biology, Sydney, NSW, Australia

[2] Sydney Medical School, University of Sydney, Sydney, NSW, Australia

[3] Gastroenterology and Liver Research Laboratory, Ingham Institute, Liverpool, NSW, Australia

[4] Australian National University, Canberra, ACT, Australia

[5] Storr Liver Centre, Westmead Institute for Medical Research, University of Sydney and Westmead Hospital, Westmead, NSW 2145, Australia

[6] A.W. Morrow Gastroenterology and Liver Centre, Royal Prince Alfred Hospital, Sydney, NSW, Australia

[7] Department of Infectious Diseases, Molecular Virology, Heidelberg University Hospital, Heidelberg, Germany

[8] South-Western Sydney Clinical School, University of New South Wales, Liverpool, NSW, Australia

[9] Department of Gastroenterology and Hepatology, Liverpool Hospital, Liverpool, NSW, Australia

[10] German Center for Infection Research (DZIF), Heidelberg Partner Site, Heidelberg, Germany

Correspondence

Nicholas Shackel, Gastroenterology and Liver Research Group, Ingham Institute, University of New South Wales, via PO Box 3151 (Westfields Liverpool), Liverpool NSW 2170, Australia; Email: n.shackel@unsw.edu.au

Supplementary Figure 1

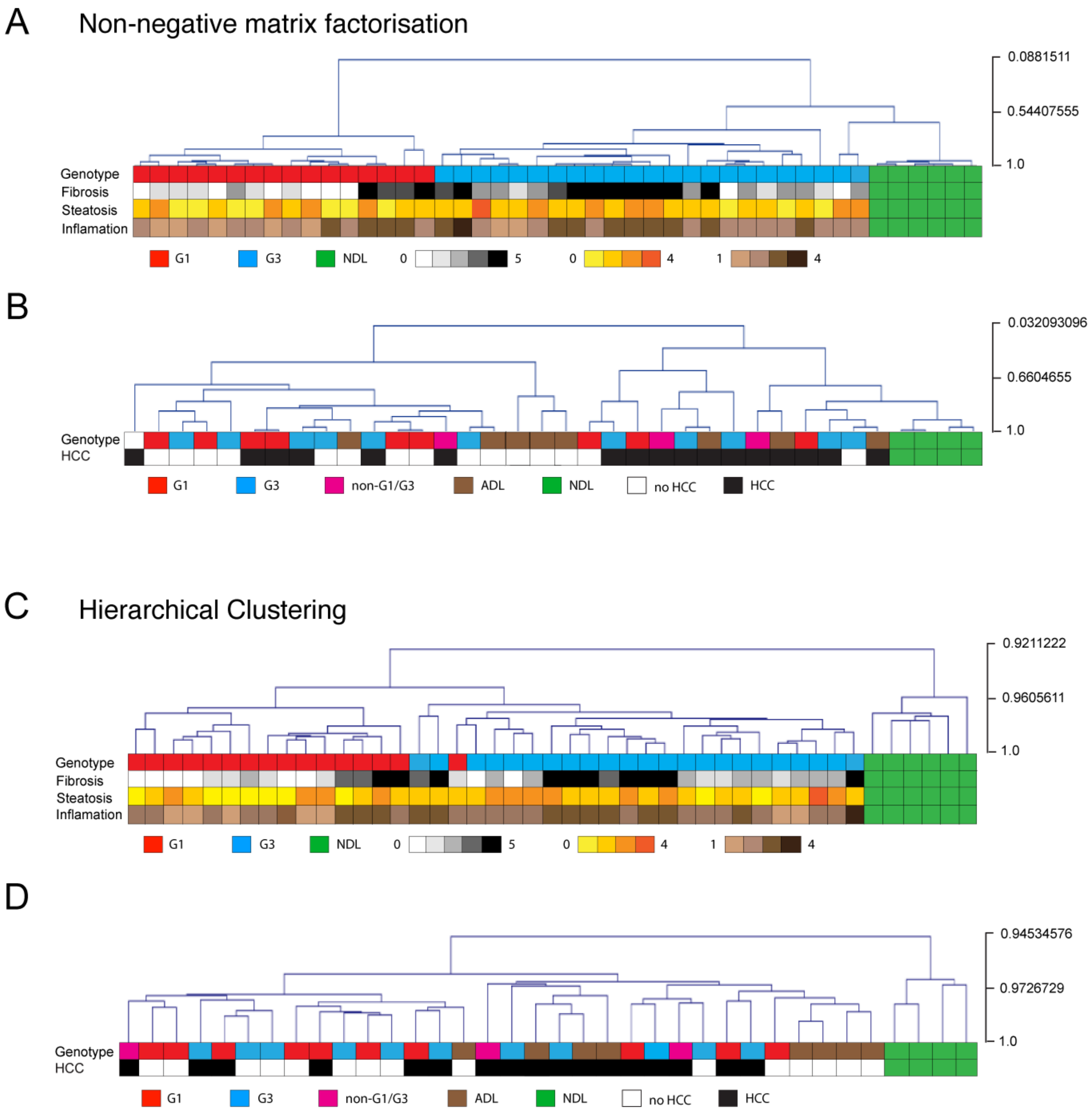

Supplementary Figure 2

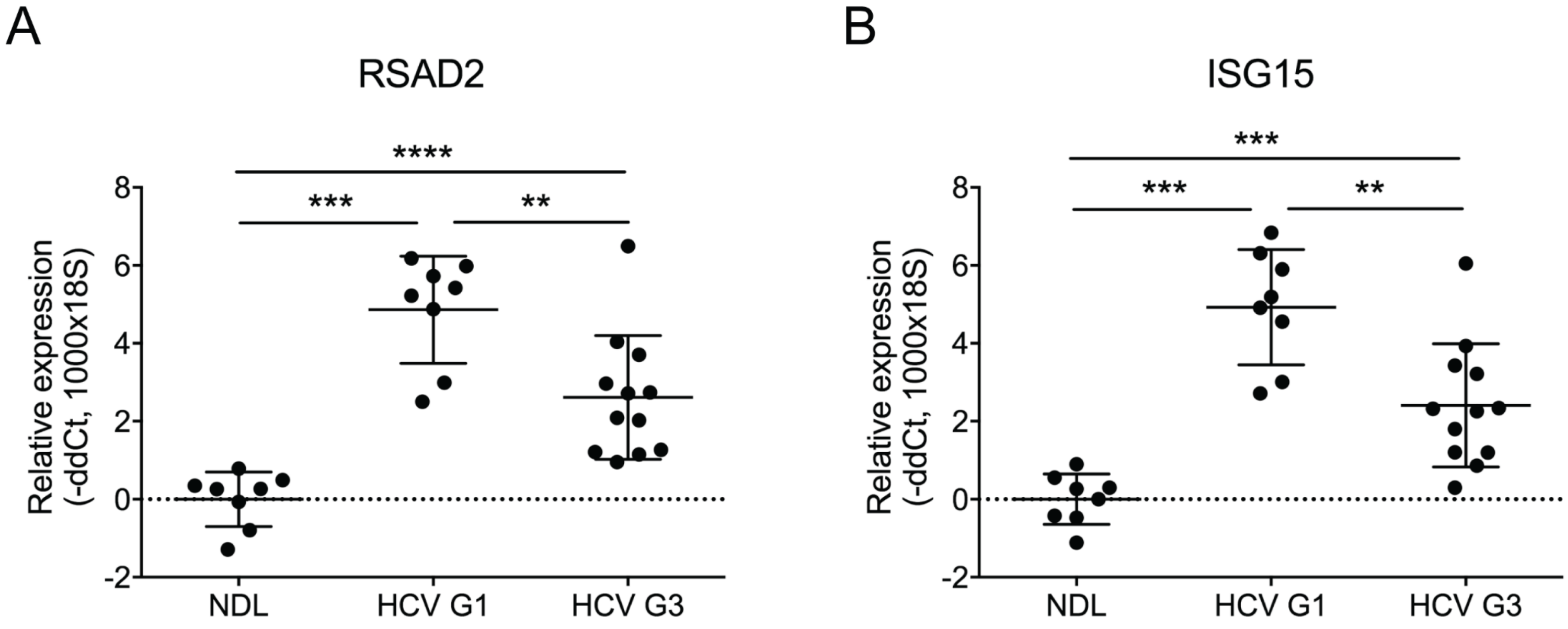

Supplementary Figure 3

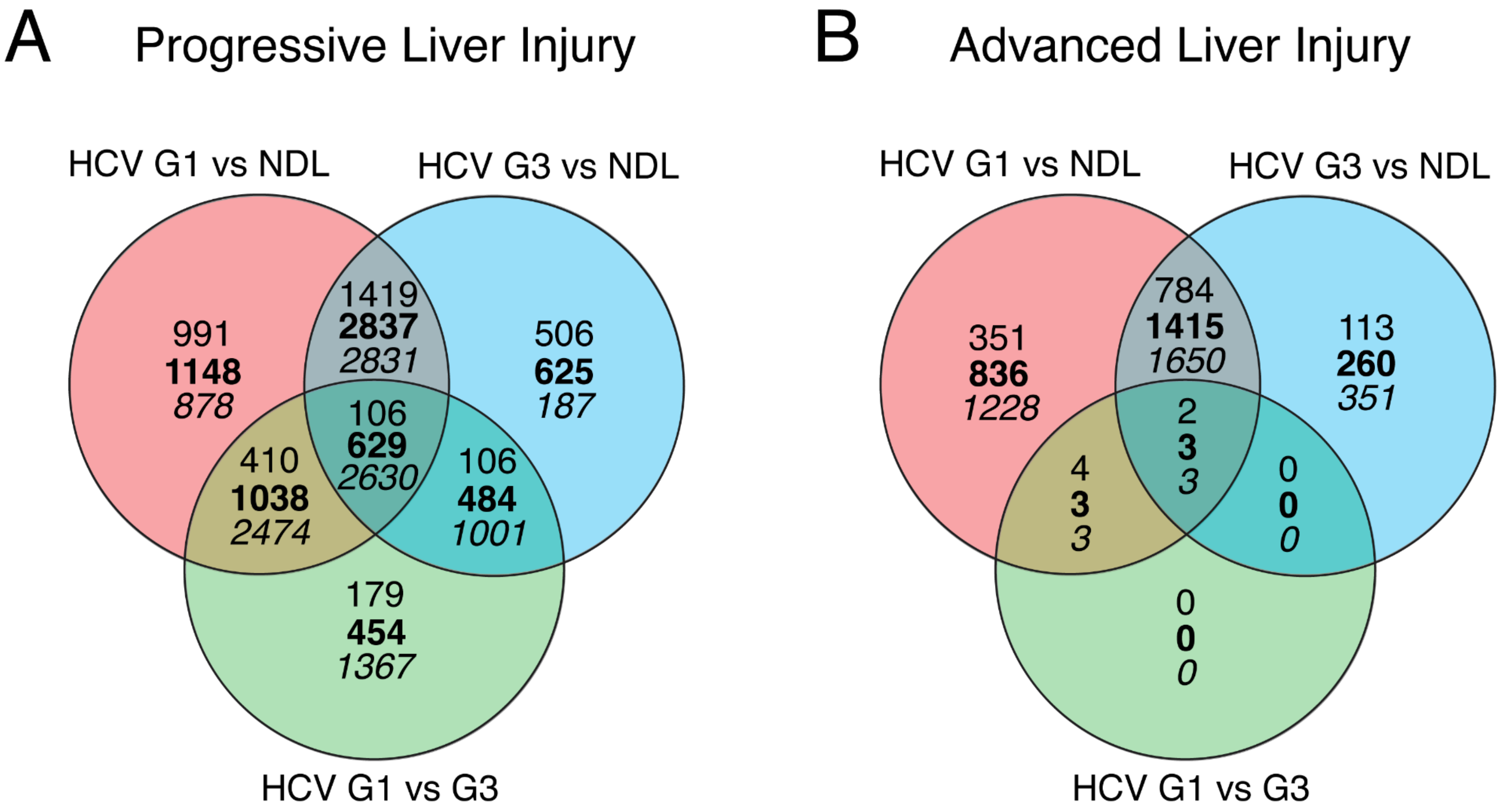

Normal > 2-fold
Bold > 1.5-fold
Italics No cut-off

Supplementary Figure legends

Supplementary Figure 1 – Unsupervised analysis of liver genes expression in HCV liver injury

Non-negative matrix factorisation (A-B) and hierarchical clustering (C-D) of the whole liver gene expression profiles of HCV-induced progressive liver injury (A, C) and advanced liver injury (B, D). In progressive liver injury (A, C) 3 groups shows distinct separation: patients infected with HCV Genotype 1 (red); genotype 3 (blue) and non-diseased liver (green). This separation is not observed in advanced liver injury (B, D).

Supplementary Figure 2 – ISGs are upregulated in HCV-infected tissues

Taqman qRT-PCR validation of interferon stimulated genes RSAD2 and ISG15 in progressive HCV-induced liver disease comparing genotype 1 (HCV G1, n=8), genotype 3 (HCV G3, n=12), and non-diseased liver (NDL, n=8); Mann-Whitney *U*-test; **p<0.01, ***p<0.001, ****p<0.0001.

Supplementary Figure 3 - Overlap of differentially expressed genes between non-diseased livers (NDL) and livers from patients infected with HCV genotype 1 (HCV G1) or genotype 3 (HCV G3)

Venn diagrams show the overlap of differentially expressed liver genes in HCV-induced progressive (A) and advanced (B) liver injury. Numbers refer to genes thresholded with a fold change of >2 (normal font), of >1.5 (bold), and with no threshold (italics).

Supplementary Tables

Supplementary Table 1 – Top 100 up- and down-regulated genes in progressive HCV liver injury (HCV genotype 3 vs genotype 1).

	Increased expression in HCV genotype 3			Decreased expression in HCV genotype 3		
**Rank**	**Gene symbol**	**Fold Change**	**p-value***	**Gene symbol**	**Fold Change**	**p-value***
1	STMN2	4.61	0.031	CROP	-14.68	<0.001
2	MMP7	4.22	<0.001	ASCL1	-14.36	0.042
3	ACTG2	4.01	0.478	C1orf63	-14.08	<0.001
4	AKR1B10	3.95	0.379	PILRB	-13.75	<0.001
5	CLOCK	3.85	<0.001	AFAR3	-13.40	<0.001
6	SPINK1	3.68	0.164	AHSA2	-11.36	<0.001
7	HLA-DQB2	3.67	0.017	RAPH1	-11.31	<0.001
8	IGJ	3.53	<0.001	CDK5RAP3	-10.32	<0.001
9	SAA2	3.45	0.053	DNAH1	-10.25	<0.001
10	C15orf48	3.42	<0.001	GSDML	-9.23	<0.001
11	RBM43	3.37	<0.001	FAM99A	-9.00	<0.001
12	CLDN11	3.30	<0.001	NKTR	-8.59	<0.001
13	SCGB3A1	3.24	0.006	CAPN12	-8.13	<0.001
14	SAA1	3.20	0.070	LOC440354	-8.11	<0.001
15	CTSG	3.20	<0.001	WDR33	-8.07	<0.001
16	COL1A1	3.12	<0.001	HAPLN4	-7.87	<0.001
17	SPP1	3.11	0.012	RBM33	-7.76	<0.001
18	ITM2A	3.05	<0.001	LOC440993	-7.68	<0.001
19	MYH11	3.05	0.026	PLA2G4B	-7.36	<0.001
20	THY1	2.97	<0.001	ATHL1	-7.28	<0.001
21	TMEM119	2.93	<0.001	PRPF4B	-7.25	<0.001
22	CD24	2.91	0.012	LOC285908	-6.85	<0.001
23	CLDN10	2.87	<0.001	PNN	-6.82	<0.001
24	FCER1A	2.86	<0.001	LOC387601	-6.74	<0.001
25	MOXD1	2.82	0.012	NSUN5C	-6.71	<0.001
26	LYZ	2.81	0.007	CHRD	-6.54	<0.001
27	CTSC	2.81	<0.001	C6orf111	-6.39	<0.001
28	FABP4	2.80	<0.001	LOC149134	-6.37	<0.001
29	ENTPD1	2.80	<0.001	LRCH4	-6.33	<0.001
30	S100A11	2.76	<0.001	LRP5L	-6.31	<0.001
31	HLA-DPB1	2.73	<0.001	SCNN1D	-6.21	<0.001
32	NTS	2.73	<0.001	SMG1	-6.18	<0.001
33	CTHRC1	2.73	<0.001	C20orf59	-6.16	<0.001
34	TIMD4	2.71	<0.001	AFG3L1	-6.16	<0.001
35	LCN2	2.69	<0.001	HEMK1	-6.01	<0.001
36	CXCR3	2.68	<0.001	NPHP3	-5.87	<0.001
37	SPON1	2.68	0.012	MAPK8IP3	-5.67	<0.001
38	CD248	2.66	<0.001	LOC440503	-5.60	<0.001
39	GPC4	2.64	<0.001	WBSCR14	-5.54	<0.001

40	QPCT	2.62	<0.001	CRYGS	-5.48	<0.001
41	RGS10	2.62	<0.001	KIAA1875	-5.42	<0.001
42	SAA1	2.62	0.042	ONECUT1	-5.30	<0.001
43	TIMP2	2.61	<0.001	ZDHHC11	-5.25	<0.001
44	SLAMF8	2.61	<0.001	C1orf188	-5.24	<0.001
45	KLRB1	2.61	<0.001	HNRPDL	-5.23	<0.001
46	KRT19	2.59	<0.001	NPIP	-5.07	<0.001
47	GLIPR1	2.58	<0.001	LOC440348	-5.02	<0.001
48	FHL2	2.57	<0.001	CAPN3	-4.86	<0.001
49	FBLN1	2.57	<0.001	IFRD1	-4.86	<0.001
50	VPS24	2.56	<0.001	GABBR1	-4.85	<0.001
51	FPR1	2.56	<0.001	MRPS25	-4.82	<0.001
52	GAS1	2.54	<0.001	CCDC14	-4.81	<0.001
53	JAM2	2.53	<0.001	EPB41L5	-4.74	<0.001
54	DKK3	2.53	<0.001	XIST	-4.71	0.017
55	APOD	2.52	0.143	C9orf45	-4.70	<0.001
56	SMOC2	2.52	0.012	TRPV1	-4.70	<0.001
57	FXYD2	2.52	<0.001	HSD17B3	-4.67	<0.001
58	ANXA2	2.51	<0.001	WSB1	-4.60	<0.001
59	COG5	2.50	<0.001	VAMP1	-4.59	<0.001
60	EMP1	2.50	0.007	CYP3A43	-4.58	<0.001
61	FAM26F	2.50	0.026	COL27A1	-4.56	<0.001
62	CD48	2.49	<0.001	C8orf46	-4.56	<0.001
63	RPS4Y1	2.49	<0.001	ATG16L2	-4.53	<0.001
64	IL8	2.48	0.266	DIDO1	-4.49	<0.001
65	RNASE6	2.48	<0.001	LOC88523	-4.49	<0.001
66	PRKCDBP	2.48	<0.001	GCN5L2	-4.46	<0.001
67	DCN	2.48	<0.001	C1orf168	-4.45	<0.001
68	PLAC9	2.48	<0.001	FNBP4	-4.45	<0.001
69	LGALS3	2.46	<0.001	CCDC71	-4.43	<0.001
70	COL1A2	2.45	<0.001	KIFC2	-4.39	<0.001
71	KRT7	2.44	<0.001	ZNF789	-4.38	<0.001
72	OSTbeta	2.44	0.070	KLHDC1	-4.36	<0.001
73	DCN	2.44	<0.001	CLK1	-4.30	<0.001
74	NRXN2	2.43	0.006	MYH3	-4.29	<0.001
75	VTCN1	2.42	0.135	DMTF1	-4.27	<0.001
76	TSPAN13	2.42	<0.001	CRIPAK	-4.27	<0.001
77	PLAUR	2.42	<0.001	WDR27	-4.23	<0.001
78	PMEPA1	2.42	<0.001	RBM6	-4.18	<0.001
79	MMP9	2.41	0.070	NRBP2	-4.18	<0.001
80	CLIC6	2.41	0.044	SNORA70	-4.16	<0.001
81	CXCL6	2.40	0.017	RAB24	-4.14	<0.001
82	GPNMB	2.40	<0.001	TTLL3	-4.12	<0.001
83	SPRY1	2.39	<0.001	NGFRAP1	-4.11	<0.001
84	CPA3	2.38	0.006	STAG3L1	-4.06	<0.001
85	EFEMP1	2.38	0.044	PHACS	-4.05	<0.001
86	EMP3	2.37	<0.001	PTBP2	-4.04	<0.001
87	PODN	2.37	<0.001	RAPH1	-3.97	<0.001
88	FRZB	2.35	<0.001	COL7A1	-3.97	<0.001

89	PLA2G7	2.35	<0.001	NFKBIZ	-3.96	<0.001
90	CCL4L1	2.34	<0.001	MGC33556	-3.93	<0.001
91	AIF1	2.33	<0.001	SFRS14	-3.93	<0.001
92	RNASE1	2.32	<0.001	XAF1	-3.92	<0.001
93	IGFBP6	2.32	<0.001	DDX17	-3.87	<0.001
94	CLDN10	2.32	<0.001	INDOL1	-3.87	<0.001
95	HSPB8	2.32	<0.001	C17orf55	-3.85	<0.001
96	EGR1	2.32	0.053	TDRD6	-3.84	<0.001
97	SYT13	2.32	0.031	MT1E	-3.82	<0.001
98	CST7	2.31	<0.001	SLC25A27	-3.82	<0.001
99	FBLN1	2.31	0.007	JOSD3	-3.75	<0.001
100	NQO1	2.31	0.012	FAM76B	-3.74	<0.001

* FDR adjusted p-value (Benjamini-Hochberg)
